# Transcriptional signatures of Zika virus infection in astrocytes

**DOI:** 10.1007/s13365-020-00931-3

**Published:** 2021-01-06

**Authors:** Blake Schouest, Tiffany A. Peterson, Dawn M. Szeltner, Elizabeth A. Scheef, Melody Baddoo, Nathan Ungerleider, Erik K. Flemington, Andrew G. MacLean, Nicholas J. Maness

**Affiliations:** 1 Tulane National Primate Research Center, Tulane University, Covington, LA USA; 2 Biomedical Sciences Training Program, Tulane University School of Medicine, New Orleans, LA USA; 3 Department of Pathology and Laboratory Medicine, Tulane University School of Medicine, New Orleans, LA USA; 4 Department of Microbiology and Immunology, Tulane University School of Medicine, New Orleans, LA USA

**Keywords:** Zika virus, Astrocytes, Nonhuman primates, RNA sequencing

## Abstract

Astrocytes are an early and important target of Zika virus (ZIKV) infection in the developing brain, but the impacts of infection on astrocyte function remain controversial. Given that nonhuman primate (NHP) models of ZIKV infection replicate aspects of neurologic disease seen in human infections, we cultured primary astrocytes from the brain tissue of infant rhesus macaques and then infected the cells with Asian or African lineage ZIKV to identify transcriptional patterns associated with infection in these cells. The African lineage virus appeared to have greater infectivity and promote stronger antiviral signaling, but infection by either strain ultimately produced typical virus response patterns. Both viruses induced hypoxic stress, but the Asian lineage strain additionally had an effect on metabolic and lipid biosynthesis pathways. Together, these findings describe an NHP astrocyte model that may be used to assess transcriptional signatures following ZIKV infection.

## Introduction

For decades following the initial isolation of Zika virus (ZIKV) from a sentinel rhesus macaque in Uganda in 1947 (Dick et al. 1952), ZIKV was not considered a significant human pathogen (Gubler et al. 2017). The Pacific island outbreaks in 2007 and 2013 changed the perception that ZIKV could not cause widespread outbreaks or significant human disease (Musso and Gubler 2016), outcomes which are now well recognized following the recent epidemics. Despite these epidemiological patterns, analyses comparing Asian lineage viruses, which are responsible for modern outbreaks, with ancestral African lineage viruses have paradoxically found higher virulence in African isolates (Simonin et al. 2017). Virus response patterns are influenced not only by the phylogeny of the infecting virus but also by host factors, therefore, well-chosen models are necessary to identify the factors contributing to neurovirulence in different ZIKV strains.

Among the animal models that rapidly emerged to study ZIKV viral dynamics and antiviral immune responses, nonhuman primates (NHPs) have held particular interest because macaques replicate aspects of neurologic diseases observed in human infections (Adams Waldorf et al. 2016; Coffey et al. 2018; Nguyen et al. 2017). Additionally, infant macaques have been utilized to assess neurodevelopmental effects of ZIKV infection (Mavigner et al. 2018) and gauge the ability of maternally acquired immunity to protect from postnatal challenge (Maness et al. 2019). Given that astrocytes are an early and important target of ZIKV in the developing brain (Potokar et al. 2019; van den Pol et al. 2017), we hypothesized that astrocytes from infant macaques would be susceptible to ZIKV infection and that these cells may be amenable to an in vitro culture system that could be used to explore transcriptional signatures associated with ZIKV infection.

## Materials and methods

### Primary astrocyte culture and infection

Brain tissue was collected from 3 Indian-origin, infant rhesus macaques (IT, MF, and MH) at Tulane National Primate Research Center. The animals utilized were naïve to ZIKV at the time of euthanasia, which was approximately at 9 months of age, and methods for euthanasia were consistent with the recommendations of the American Veterinary Medical Association's Panel on Euthanasia. Cortical tissue was collected into Dulbecco’s modified Eagle medium: Nutrient Mixture F-12 (DMEM/F-12, Gibco) and mechanically dissociated and treated with 0.25% trypsin and 0.1% DNase for 1 h at 37 °C with agitation every 10 min. After centrifugation at 1600 rpm for 5 min, cells were resuspended in DMEM/F-12 and passed through a ~ 120 μm filter. Cells were centrifuged at 1600 rpm for 5 min, resuspended in DMEM/F-12 supplemented with 1X Antibiotic–Antimycotic (Gibco) and 0.5 ng/ml granulocyte-macrophage colony stimulating factor (GM-CSF), and cultured in T75 flasks at 37 °C and 5% CO_2_. Astrocyte cultures were left uninfected or infected in three biological replicates (IT, MF, and MH) with either Rio-U1 (KU926309; Bonaldo et al. 2016), an Asian-lineage isolate from Rio de Janeiro that was minimally passaged in Vero cells (ATCC CCL-81), or MR766 (KU963573; obtained through BEI Resources, NIAID, NIH, as part of the WRCEVA program), the reference African-lineage strain. Viral stocks were quantified using an in-house qRT-PCR assay as described previously (Magnani et al. 2018), and infections were carried out at by adding 3.2 × 10^9^ copies of Rio-U1 or 9.4 × 10^8^ copies of MR766 to each T75 flask for 24 h at 37 °C and 5% CO_2_.

### Astrocyte imaging

Separate astrocyte cultures were obtained as described above from the cortical tissue of two naïve infant rhesus macaques (animal A and animal B). For imaging experiments, astrocyte cultures were prepared in T25 flasks, and cells from each flask were detached using 0.25% trypsin-EDTA solution (Gibco) and subcultured into an 8-well Permanox chambered slide (Nunc). Twenty-four hours later, the astrocytes were infected with either Rio-U1 or MR766 or left uninfected. At 24 h post-infection, cells were fixed with 2% paraformaldehyde for 5 min and washed 2× with phosphate-buffered saline (PBS) before permeabilization with PBS-normal goat serum (NGS, 10%)-fish skin gelatin (FSG, 0.2%)-Triton X-100 (Tx100, 0.1%). Cells were stained for the astrocyte marker glial fibrillary acidic protein (GFAP) using the GFAP-Cy3 conjugate (Sigma, catalog # C9205) at a 1:50 dilution as per (Renner et al. 2013). Cells were also stained for viral antigens using antibodies specific for either envelope (ENV, anti-ENV monoclonal-biotin conjugate at a 1:50 dilution; secondary: streptavidin Alexa Fluor 488 conjugate (Invitrogen) at a 1:1000 dilution) or nonstructural protein 3 (NS3, dengue virus mouse anti-NS3 LifeSpan Bio LS-C348874 at a 1:100 dilution; secondary: goat anti-mouse IgG Alexa Fluor 488 conjugate (Invitrogen) at a 1:1000 dilution). We previously found the dengue virus NS3 antibody to cross-react with ZIKV NS3. After staining, chambers were detached, and slides were mounted using ProLong Gold Antifade Mountant with DAPI (Invitrogen), cured, and imaged at 40× magnification using a Nikon Eclipse Ti-2 microscope. Images were edited using Fiji (ImageJ version 2.0.0-rc-69/1.52p) open-source image processing software. Quantification of positive labeling was completed by analyzing ten nonoverlapping high-power fields (40×) for each stain. DAPI positive nuclei were counted for total cell count, and GFAP, ENV, and NS3 positively labeled cells were counted and percentages of singly and doubly positively labeled cells were calculated.

### RNA sequencing and analysis

Among three biological replicates and three conditions each (uninfected or infected with Rio-U1 or MR766), astrocyte cultures were lysed, and total RNA was extracted using the RNeasy Mini Kit (Qiagen) and purified using the RNA Clean and Concentrator Kit (Zymo Research). BGI Genomics carried out mRNA enrichment using oligo dT-based selection, library preparation, and paired-end RNA sequencing (RNAseq) on a BGISeq instrument.

Sequencing data were analyzed using an in-house pipeline. Briefly, reads were deduplicated using the BBMap package clumpify and mapped to the rhesus macaque transcriptome (Ensembl Mmul_10 assembly) using kallisto (Bray et al. 2016). DESeq2 (Love et al. 2014) was used with default parameters to calculate differentially expressed genes (DEGs) with an adjusted *p* < 0.05 in astrocytes infected with either virus relative to uninfected controls. Pathway analysis was carried out using gene set variation analysis (GSVA) (Hänzelmann et al. 2013), gene set enrichment analysis (GSEA) (Subramanian et al. 2005), and ReactomePA (Yu and He 2016). For GSVA analysis, gene sets in the Reactome database were used, and pairwise comparisons among conditions were carried out using limma (Ritchie et al. 2015). Gene sets were considered significantly differentially enriched at *p* < 0.05. For GSEA analysis, a false discovery rate (FDR) below 25% was used to identify gene sets in the Hallmarks, Reactome, and Gene Ontology (GO) collections that were significantly enriched at 3 or 7 days post-infection (dpi) relative to pre-infection. For these analyses, a gene set permutation of 1000 was utilized. For ReactomePA, pathway analysis was carried out on DEGs identified by sleuth to identify enriched gene networks at *p* < 0.05. Volcano plots, heatmaps, and Venn diagrams were generated using the EnhancedVolcano, pheatmap, and VennDiagram packages in R, respectively. For heatmaps and scatterplots of read count data, log2-transformed read counts of genes responsible for core enrichment of the indicated gene sets are plotted.

## Results

### ZIKV infectivity and antiviral response patterns

**Fig. 1:**
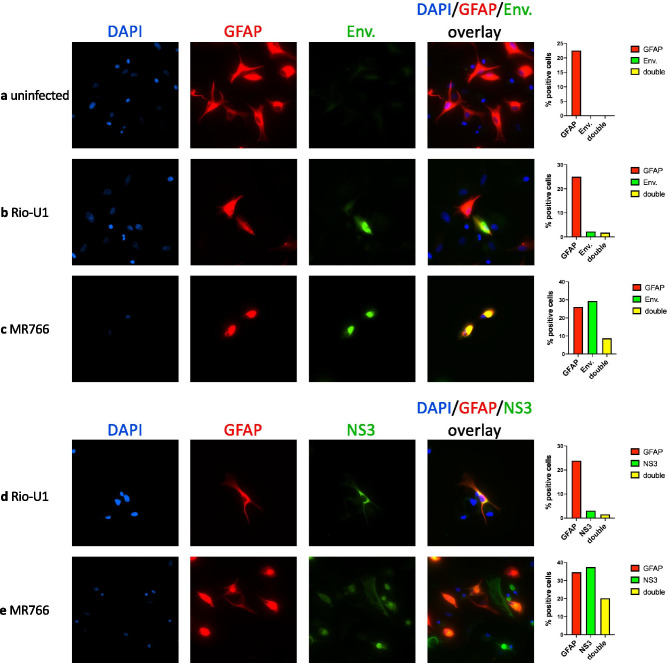
Macaque astrocytes are susceptible to Asian- and African-lineage ZIKV infection. Immunofluorescence images (40×) of primary astrocytes isolated and cultured from the brain tissue of a naïve infant rhesus macaque (animal A). Astrocytes were either left uninfected **a** or infected with the Asian-lineage strain Rio-U1 **b** and **d** or the African-lineage strain MR766 **c** and **e** and stained at 3 dpi for the astrocyte marker GFAP-CY3 in red **a**–**e**, the viral proteins envelope (ENV) **a**–**c** or nonstructural protein 3 (NS3) **d**–**e** in green, and the cell nucleus by DAPI in blue. Double labeled cells with both GFAP-CY3 and viral protein-positive labeling appear yellow (overlay image, rightmost column). For quantification, 10-high-power-fields were used to count DAPI positive nuclei for total cells and calculate percentages of GFAP, ENV, or NS3 positive cells as well as double-labeled GFAP/ENV or GFAP/NS3

Twenty-four hours following infection with either Asian (Rio-U1) or African (MR766) lineage ZIKV, cultured astrocytes (GFAP+) from infant macaques were co-positive for the viral antigens envelope (E) and nonstructural protein 3 (NS3), indicating permissiveness to infection (Figs. 1a–e and S1a-e). NS3 staining indicates active viral replication, as NS3 is the viral protease and helicase. Despite the similar number of viral copies in inoculums of MR766 and Rio-U1, a greater number of cells were infected with the African-lineage virus (Figs. 1 and S1). Although a higher proportion of cells infected with MR766 stained positively for viral antigens compared with GFAP, cultured astrocytes have lower GFAP expression than in vivo, possibly accounting for this effect. Additionally, all astrocyte cultures showed expression of several key genes relating to astrocyte development (Fig. S4).

**Fig. 2:**
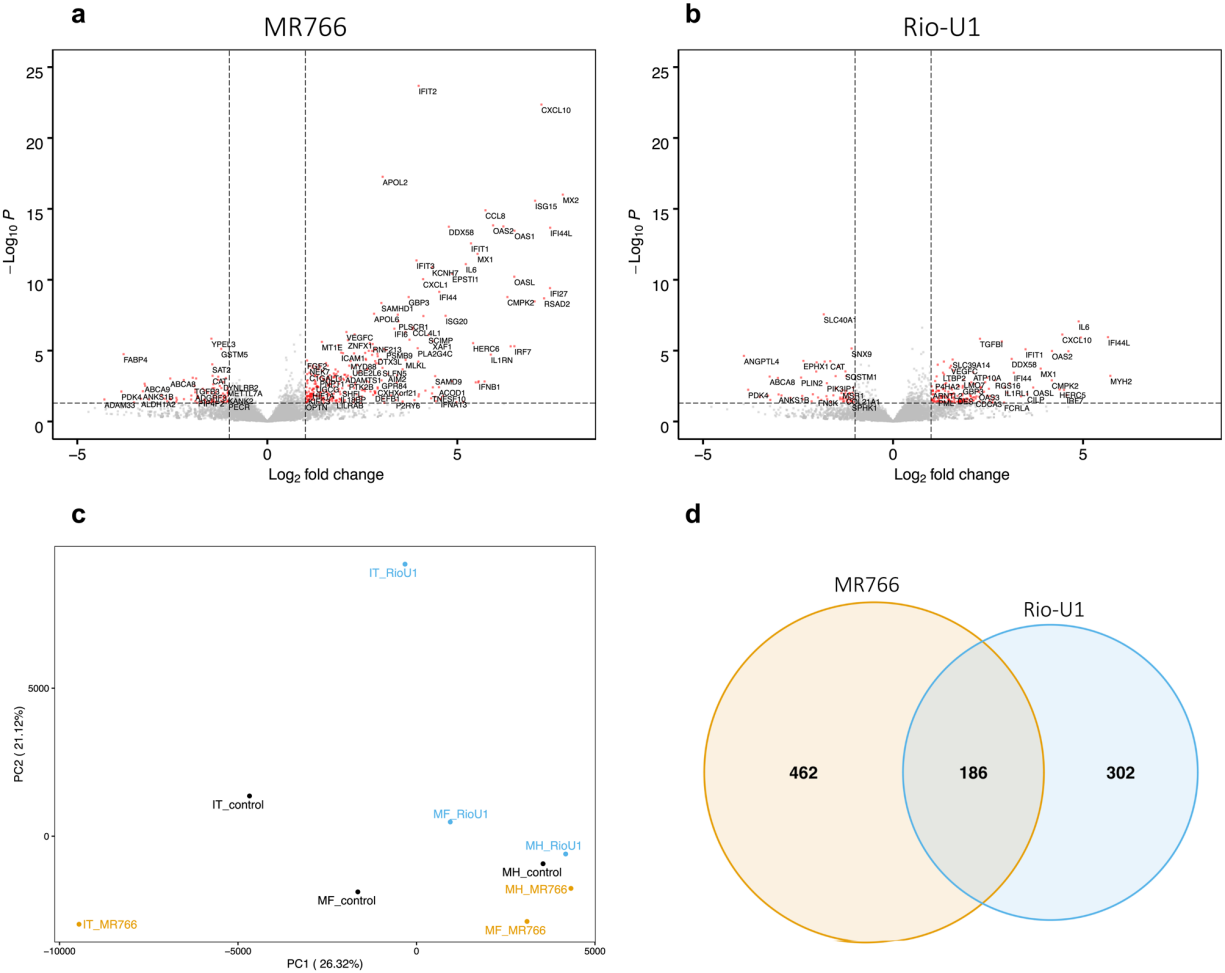
Lineage-dependent impacts on host gene expression. Volcano plots showing differentially expressed genes (DEGs) identified by RNA-seq analysis in astrocytes infected with MR766 **a** or Rio-U1 **b**. **c** PCA plot showing variance in gene expression data in astrocytes that were left uninfected (black) or infected with MR766 (yellow) or Rio-U1 (blue). IT, MF, and MH represent biological replicates. **d** Venn diagram depicting overlapping and unique DEGs in astrocytes infected with either virus

Relative to uninfected controls, astrocytes infected with MR766 also showed a greater number of differentially expressed genes compared with astrocytes infected with Rio-U1 (Fig. 2a–b), and most of these genes appeared to relate to the interferon (IFN) response and pro-inflammatory cytokine signaling. Although Rio-U1 infected cells also showed activation of genes relating to inflammation, the number of these genes and magnitude of their induction was greatest in astrocytes infected with MR766 (Figs. 2a–b and S2a-c). MR766 also affected the expression of several genes relating to lipid movement (APOL2, APOL6, FABP4, ABCA8) (Fig. 2a) and metabolism (HELZ2) (Fig. S2a). Interestingly, HELZ2 is a known restriction factor of the related flavivirus dengue virus (DENV) that mediates antiviral effects through modulation of host lipid metabolism (Fusco et al. 2017). Likewise, Rio-U1 infection affected HELZ2 expression and lipid metabolism-related genes (ANGPTL4, PDK4, EPHX), but the Asian-lineage virus also appeared to alter the maintenance of structural proteins (MYH2) and showed induction of TGFB1 (Figs. 2b and S2c), possibly pointing to an immunomodulatory phenotype. Principal component analysis (PCA) showed variation among the biological replicates (Fig. 2c), resulting in different baseline signatures. Direct comparison of differentially expressed genes (DEGs) showed a greater number of genes that were exclusively affected by MR766 infection (462 genes) compared with Rio-U1 (302 genes). However, 186 genes were similarly modulated by both viruses (Fig. 2d). Interestingly, genes that were differentially expressed by both viruses were regulated in the same direction and to a similar magnitude (Fig. S2a). Some of the genes that were similarly modulated by MR766 and Rio-U1 related to cytokine signaling (CXCL10/11, CCL8) and the IFN response (IFIT2, ISG15, OAS2, DDX58, among others) (Fig. S2a), suggesting some degree of similarity in signaling patterns following infection with either virus.

### Transcriptional signatures associated with MR766 and Rio-U1 infection

**Fig. 3:**
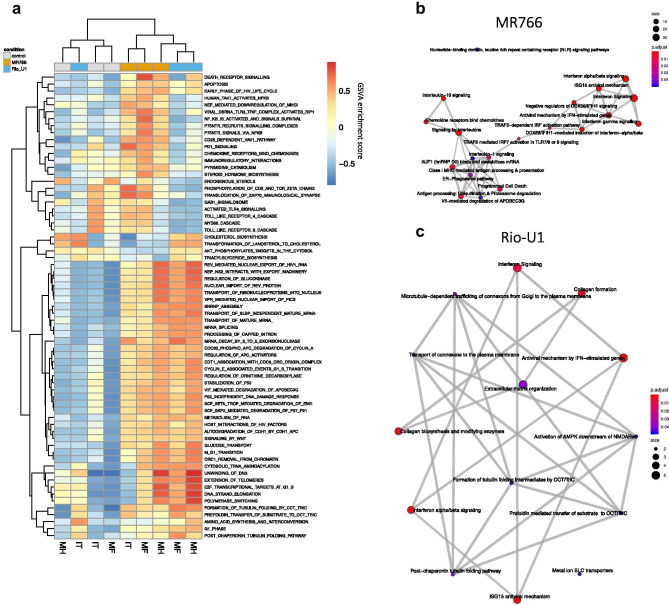
Lineage-dependent virus response patterns. **a** Heatmap showing gene set variation analysis (GAVA) enrichment scores among significantly modulated gene sets. Gene sets in the Reactome databank were included if statistical significance was attained in pairwise comparisons among conditions. Enrichment map plots showing the interrelatedness of gene set networks in astrocytes infected with MR766 **b** or Rio-U1 **c** relative to uninfected controls

Owing to variation among the biological replicates, exploratory pathway analysis was carried out using gene set variation analysis (GSVA) to survey signaling patterns among samples in a phenotype-independent manner. Hierarchical clustering of enrichment results showed activation of similar pathways in samples infected with either MR766 or Rio-U1 relative to uninfected controls, with the exception of IT infected with Rio-U1 (Fig. 3a). Both strains had an effect on the cell cycle and RNA processing that was possibly indicative of viral replication (Fig. 3a). Despite these similarities, several signaling patterns were also regulated differently among the viral strains; MR766 infected astrocytes showed induction of pathways associated with cell death, viral replication, and pathogen sensing (Fig. 3a). Given the potential for gene overlap among pathways that showed similar patterns of activation, we used ReactomePA to identify the interrelatedness of pathways induced by either virus. This analysis revealed an orchestrated IFN response in MR766 infected astrocytes involving many genes responsible for IFN signaling and response (Figs. 3b and S2b). Meanwhile, the smaller number of DEGs in Rio-U1 infected cells produced a less integrated gene expression signature with limited IFN signaling (Fig. 3c). Rio-U1 appeared to affect a separate set of functions relating to the maintenance of structural proteins, including collagen biosynthesis and tubulin folding that was unrelated to the IFN response (Figs. 3c and S2c).

**Fig. 4:**
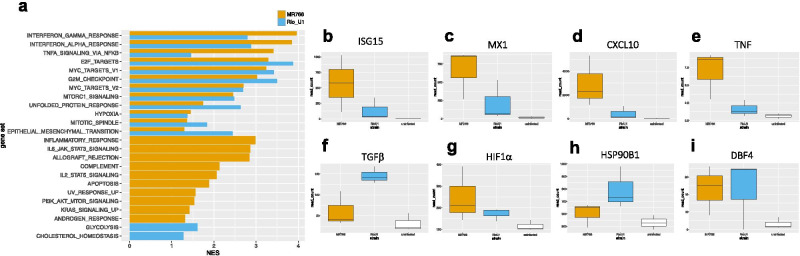
Overlapping but distinct transcriptional signatures. **a** Bar graph showing gene sets in the Hallmarks collection that were significantly enriched at FDR < 0.1 (NES normalized enrichment score). Gene sets were either similarly enriched in both viruses (top) or enriched only in MR766 (middle, yellow) or Rio-U1 (bottom, blue) infected cells. Boxplots showing normalized read counts of representative genes in MR766 (yellow) or Rio-U1 (blue) infected cells and uninfected controls (white) relating to the IFN response **b**–**c**, inflammation **d**–**e**, hypoxia signaling **f**, the unfolded protein response **g**, cell cycle arrest **h**, and immunomodulation **i**

We next turned to gene set enrichment analysis (GSEA) to directly compare phenotypes promoted by each virus relative to uninfected controls. GSEA largely replicated the patterns we observed using GSVA and ReactomePA in that MR766 and Rio-U1 both induced an antiviral IFN response and had an effect on the cell cycle (Figs. 4a and S3a-b). Again, the magnitude of IFN and inflammatory signaling was greater in MR766 infected cells (Fig. 4a), although we cannot exclude the possibility that this finding might be attributed to greater infectivity of this strain. These signaling patterns were represented at the gene level by greater read counts of IFN responsive genes such as ISG15 and MX1 and chemokines including CXCL10 and TNF (Fig. 4b–e). MR766 but not Rio-U1 promoted cell death through caspase signaling (Figs. 4a and S3c), but again, the lack of cell death signaling in Rio-U1 infected cells might be due to the lower infectivity of this virus. In addition to confirming signaling patterns that were highlighted in previous analyses, GSEA also showed an effect of Rio-U1 infection on metabolic pathways relating to cell respiration and lipid metabolism (Figs. 4a and S3e-g), pathways similar to those we observed in a cohort of pregnant rhesus macaques also infected with Rio-U1 (Schouest et al. 2020, preprint). Rio-U1 also upregulated the immunomodulatory cytokine TGFβ (Fig. 4f), and interestingly, both viruses induced AXL, a putative entry receptor for ZIKV (Fig. S3d) (Meertens et al. 2017). Finally, we identified the activation of pathways consistent with cell stress following infection with either virus. Both strains promoted the enrichment of gene sets relating to endoplasmic reticulum (ER) stress and low oxygen availability, including hypoxia, the unfolded protein response (UPR), and mTOR signaling (Fig. 4a). HIF1α, which is a key regulator of transcriptional signaling in hypoxic conditions (Majmundar et al. 2010), was induced following infection with either virus (Fig. 4g), as were HSP90B1 and DBF4 (Fig. 4h–i), which are mediators of the unfolded protein response (Rachidi et al. 2015) and cell cycle arrest (Guo et al. 2005), respectively.

**Fig. 5:**
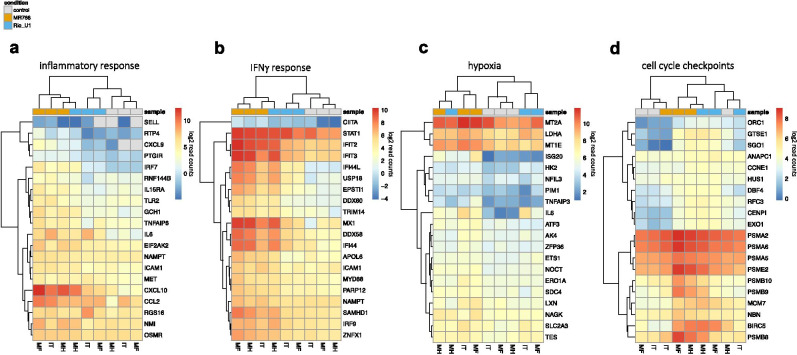
Gene-level analysis of key biological functions. Heatmaps showing log2-transformed read counts of genes involved in inflammation **a**, IFNγ signaling **b**, hypoxia signaling **c**, and cell cycle checkpoints **d**. For each heatmap, the top 20 genes responsible for core enrichment of the indicated sets from GSEA are displayed

The gene-level analysis revealed highly induced genes responsible for core enrichment of functions from GSEA, and hierarchical clustering showed strain-dependent effects on many of these signaling pathways. Inflammation and IFNγ signaling, which were among the most highly enriched pathways in MR766 infected cells, were driven by increases in the expression of chemokines (CXCL10, CCL2, CXCL9, IL6) and chemokine receptors (OSMR, IL15RA), together with molecules involved in cell adhesion (ICAM1, SELL), pathogen sensing (TLR2, DDX58), and IFN cascades (IRF7/9, IFIT1/2, TRIM14, STAT1) (Fig. 5a–b). Hypoxia was promoted by an increase in the expression of transcription factors (ATF3, ETS1) and genes relating to intracellular homeostasis (HK2, MT2A, SLC2A3) (Fig. 5c). Both viruses also appeared to have an effect on cell proliferation, with the induction of genes relating to cell cycle checkpoints such as proteasome subunits (PSMB8, PSMA2, PSMB9), DNA damage sensors (NBN, HUS1, EXO1), and microtubule/kinetochore regulators (GTSE1, CENPI) (Fig. 5d).

## Discussion

Astrocytes are an early and important target of ZIKV infection in the developing brain (Potokar et al. 2019; van den Pol et al. 2017), but the impacts of infection on cellular processes are not fully resolved. Given that astrocytes are permissive to both Asian and African ZIKV strains (Hamel et al. 2017; Meertens et al. 2017), and considering the myriad supportive roles of astrocytes in synapse maintenance and central nervous system homeostasis, infection of these cells could have multifaceted and detrimental outcomes (Potokar et al. 2019). Conflicting reports argue that ZIKV infection either increases (Stefanik et al. 2018) or decreases (Chen et al. 2018; Meertens et al. 2017) the production of proinflammatory cytokines by astrocytes, but phylogenetic differences among ZIKV strains often complicate the direct comparison of these studies (Beaver et al. 2018; Jorgačevski et al. 2019; Zhang et al. 2016). While modern Asian-lineage strains are primarily linked to neurologic diseases, studies in cell culture and in animal models have paradoxically found that infection by African-lineage ZIKV results in greater infectivity, virus production, and antiviral responses (reviewed in (Simonin et al. 2017)). Thus, we asked whether cultured astrocytes from a relevant animal model could serve as a model system to explore the effects of ZIKV infection on the transcriptome.

Flaviviruses, including ZIKV, characteristically have broad tissue tropisms (Miner and Diamond 2017), so it was not surprising that macaque astrocytes were permissive to both Asian and African lineage ZIKV infection, as has been shown in human cells (Stefanik et al. 2018). MR766 showed greater infectivity in cultured macaque astrocytes compared with Rio-U1, which was not unexpected given similar previous analyses comparing representative viruses in the Asian and African lineages (Simonin et al. 2017). MR766 is neurologically adapted owing to its extensive passage history by intracerebral inoculation of mice, which caused a deletion in the N-glycosylation motif of the envelope protein (Myrna C. Bonaldo, personal communication), perhaps making this virus uniquely predisposed to neurotropic and transcriptional outcomes. Although it is possible that a covert infection by Asian-lineage strains might permit these viruses to avoid immune detection and persist in neural tissues where they have damaging effects (Simonin et al. 2016), the higher virulence of MR766 could reflect the passage history of MR766 specifically and may not be generalizable to other African-lineage viruses. We also detected higher magnitude IFN responses in astrocytes infected with the African lineage virus, which may be directly attributed to the higher infectivity we observed in this strain. Transcriptome analysis of human astrocytes has outlined the crucial role of the IFN response in restricting ZIKV infection (Chen et al. 2018), and IFN functions are indispensable in controlling acute ZIKV infection in mice, as evidenced by the lethality of infection when IFN signaling is impaired (Morrison and Diamond. 2017). Accordingly, it was not surprising to observe that IFN was the most highly induced function by either virus.

Both strains also induced hypoxia and cell stress, which appeared to be mediated in part by HIF1α, an oxygen-sensitive transcription factor that is activated in several viral infections (Morinet et al. 2013). Oxygen stress enhances the infectivity and replication of several important human RNA viruses such as human immunodeficiency virus, hepatitis B and C, and the related flavivirus DENV (Frakolaki et al. 2018; Gan et al. 2017; Morinet et al. 2015), so a similar mechanism may exist in ZIKV infection. The unfolded protein response (UPR) and mTor signaling, other important hypoxia response pathways (Majmundar et al. 2010; Wouters and Koritzinsky 2008), were also activated by both ZIKV strains. The HIF, UPR, and mTor signaling pathways influence each other during hypoxia to produce changes in cell metabolism and proliferation (Wouters and Koritzinsky 2008), so future studies might address how these pathways affect viral replication and cell metabolism in astrocytes during ZIKV infection. In addition to its role in hypoxia signaling, UPR is a common outcome of ZIKV infection given the massive viral replication that occurs in the endoplasmic reticulum (ER) (Alfano et al. 2019). Although considered a homeostatic mechanism to relieve ER stress, UPR can also halt cell replication and promote apoptosis in cases of prolonged ER stress, pathways that have been observed in neural cells infected with ZIKV that are thought to contribute to microcephaly (Alfano et al. 2019). Transcriptome analysis of human astrocytes found that ZIKV promotes UPR signaling to support viral replication (Kozak et al. 2017), a mechanism that might similarly sustain infection in macaque cells.

Rio-U1 infection had an influence on metabolic pathways regulating lipid catabolism and cell respiration, functions related to autophagy that are modulated by several flaviviruses (Chiramel and Best 2018; Gratton et al. 2019). Autophagy generally functions to induce innate and adaptive immune responses during microbial infections (Gratton et al. 2019), and flaviviruses such as DENV and ZIKV interact directly with autophagy machinery to manipulate lipid content in a manner that enhances infectivity (Chiramel and Best 2018; Gratton et al. 2019; Jordan and Randall 2016).

An important contribution of the present study was replication of previously identified ZIKV-associated transcriptional patterns in primary astrocytes from infant macaques. These data should be interpreted with the caveats that a sizeable fraction of the cells following in vitro culture lacked GFAP staining and the ZIKV strains analyzed were not equally infective in these cells. It is noted that cultured astrocytes have lower levels of GFAP expression than in vivo, and all cultures showed expression of several genes relating to astrocyte development, which we feel supports the use of this model to explore signaling patterns following ZIKV infection. Although differences in infectivity complicate the direct comparison of signaling patterns following MR766 and Rio-U1 infection, it has been previously shown that African lineage ZIKV induces stronger antiviral responses (Simonin et al. 2017), and altered lipid metabolism through autophagy is a common feature of flavivirus infection (Jordan and Randall 2016). The extension of these findings in a macaque astrocyte model implies the broader relevance of these signaling patterns, although the differences in signaling patterns following infection with Asian and African lineage ZIKV requires further investigation.

## Supplementary Information

Below is the link to the electronic supplementary material.

Supplementary file1 - Supplementary Figure 1: Macaque astrocytes are susceptible to Asian- and African-lineage ZIKV infection (**A-E**) Immunofluorescence images (40x) of primary astrocytes isolated and cultured from the brain tissue of a naïve infant rhesus macaque (animal B). Astrocytes were either left uninfected (A) or infected with the Asian-lineage strain Rio-U1 (B & D) or the African-lineage strain MR766 (C & E) and stained at 3 dpi for the astrocyte marker GFAP-CY3 in red (A-E), the viral proteins envelope (ENV) (A-C) or nonstructural protein 3 (NS3) (D-E) in green, and the cell nucleus by DAPI in blue. Double labeled cells with both GFAP-CY3 and viral protein positive labeling appear yellow (overlay image, rightmost column). For quantification, 10-high-power-fields were used to count DAPI positive nuclei for total cells and calculate percentages of GFAP, ENV, or NS3 positive cells as well as double labeled GFAP/ENV or GFAP/NS3. (PDF 2160 KB)

Supplementary file2 - Supplementary Figure 2: Similarities in gene expression signatures (**A**) Bar graph showing the extent of up- and downregulation of genes that were similarly affected by either virus. Of the 186 genes that were differentially expressed by both viruses, the 50 genes with the highest level of significance in MR766 infected cells are shown. (**B-C**) Gene networks showing the overlap of genes involved in multiple signaling pathways in MR766 and Rio-U1 infected astrocytes. (PDF 365 KB)

Supplementary file3 - Supplementary Figure 3: Significantly modulated gene sets (**A-B**) Bar graphs showing gene sets in the Reactome (A) and Gene Ontology (GO) (B) collections that were significantly enriched at FDR<0.1. The top 10 gene sets that were either similarly enriched in both viruses (top) or enriched only in MR766 (middle, yellow) or Rio-U1 (bottom, blue) infected cells are displayed. (**C-D**) Boxplots showing normalized read counts for CASP4 (C) and AXL (D) in MR766 (yellow) or Rio-U1 (blue) infected cells and uninfected controls (white). (**E-G**) Bar graphs showing gene sets in the Hallmarks (E), Reactome (F), and Gene Ontology (GO) collections (G) that showed negative enrichment at FDR<0.25. (PDF 170 KB)

Supplementary file4 - Supplementary Figure 4: Expression of genes relating to astrocyte developmentScatterplot showing the read counts of several genes relating to astrocyte development (GO:0014002). (PDF 168 KB)
