# Supplementary figures and images for: Transcriptional signatures of Zika virus infection in astrocytes
Source: J Neurovirol. 2021 Jan 6;27(1):116–25. doi: 10.1007/s13365-020-00931-3 (PMC7921019; doi:10.1007/s13365-020-00931-3)

Figure S1

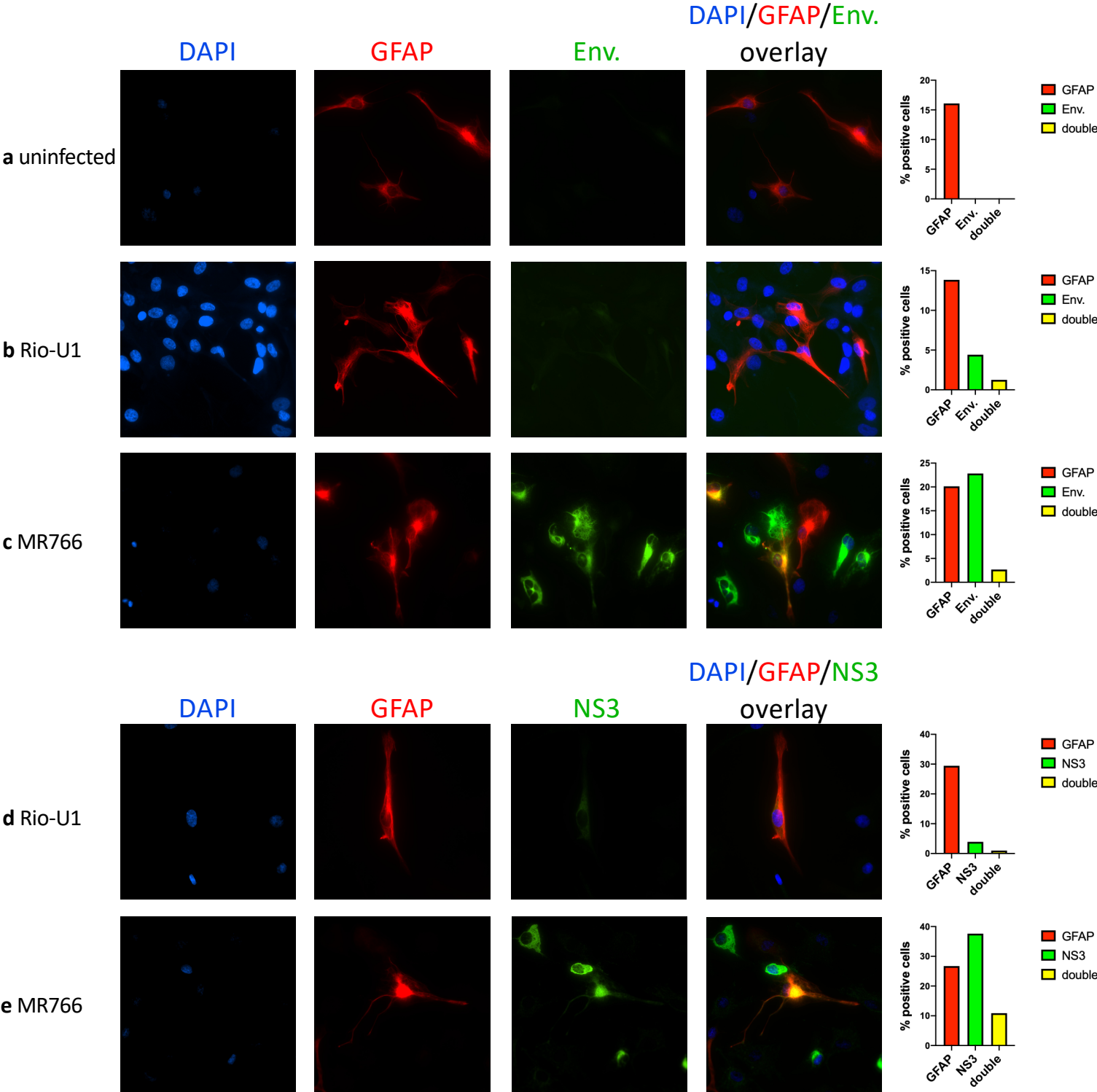

Supplement: Supplementary file 1 — Supplementary file1 - Supplementary Figure 1: Macaque astrocytes are susceptible to Asian- and African-lineage ZIKV infection (A-E) Immunofluorescence images (40x) of primary astrocytes isolated and cultured from the brain tissue of a naïve infant rhesus macaque (animal B). Astrocytes were either left uninfected (A) or infected with the Asian-lineage strain Rio-U1 (B & D) or the African-lineage strain MR766 (C & E) and stained at 3 dpi for the astrocyte marker GFAP-CY3 in red (A-E), the viral proteins envelope (ENV) (A-C) or nonstructural protein 3 (NS3) (D-E) in green, and the cell nucleus by DAPI in blue. Double labeled cells with both GFAP-CY3 and viral protein positive labeling appear yellow (overlay image, rightmost column). For quantification, 10-high-power-fields were used to count DAPI positive nuclei for total cells and calculate percentages of GFAP, ENV, or NS3 positive cells as well as double labeled GFAP/ENV or GFAP/NS3. (PDF 2160 KB) [file 13365_2020_931_MOESM1_ESM.pdf]

## Figure S2

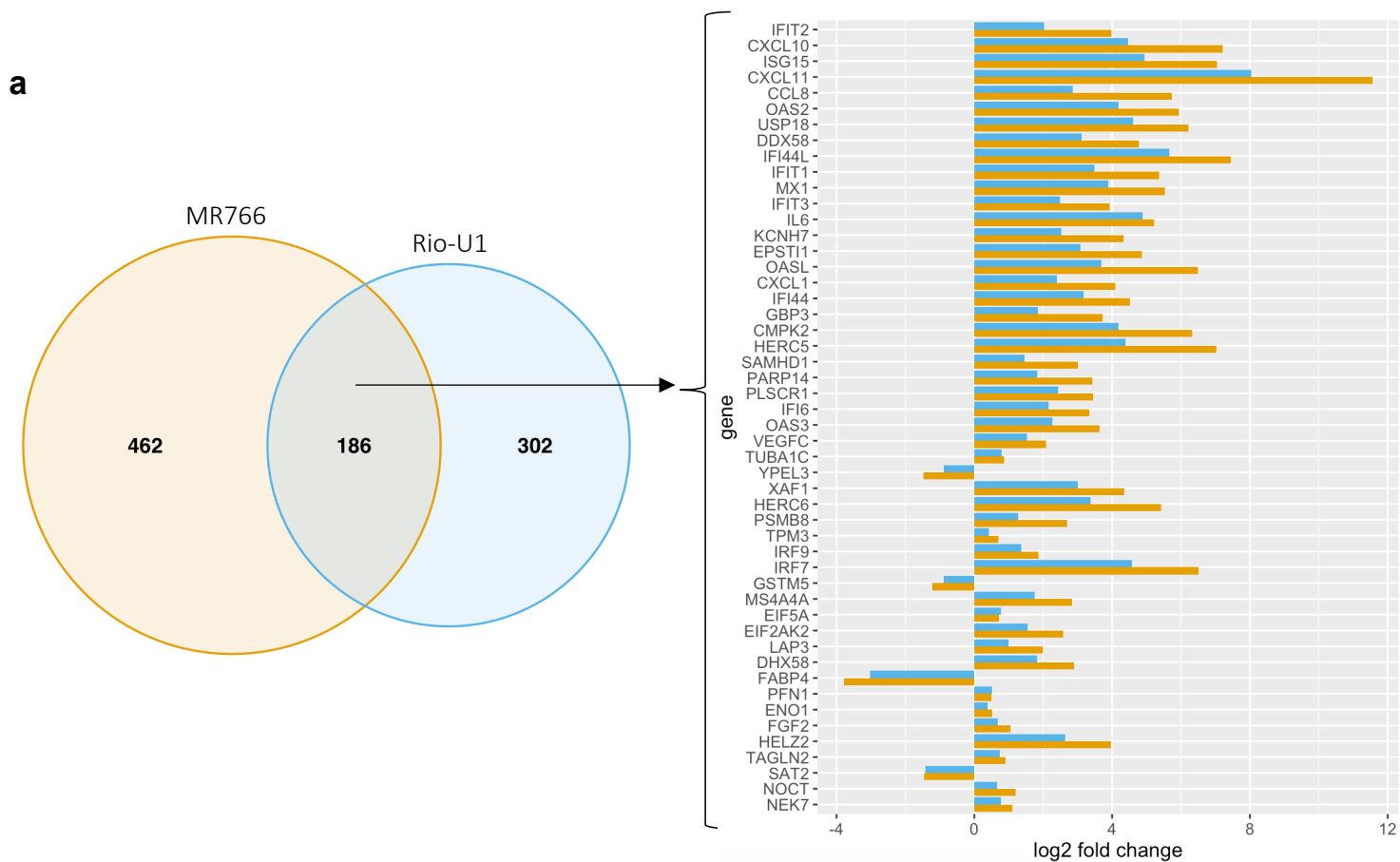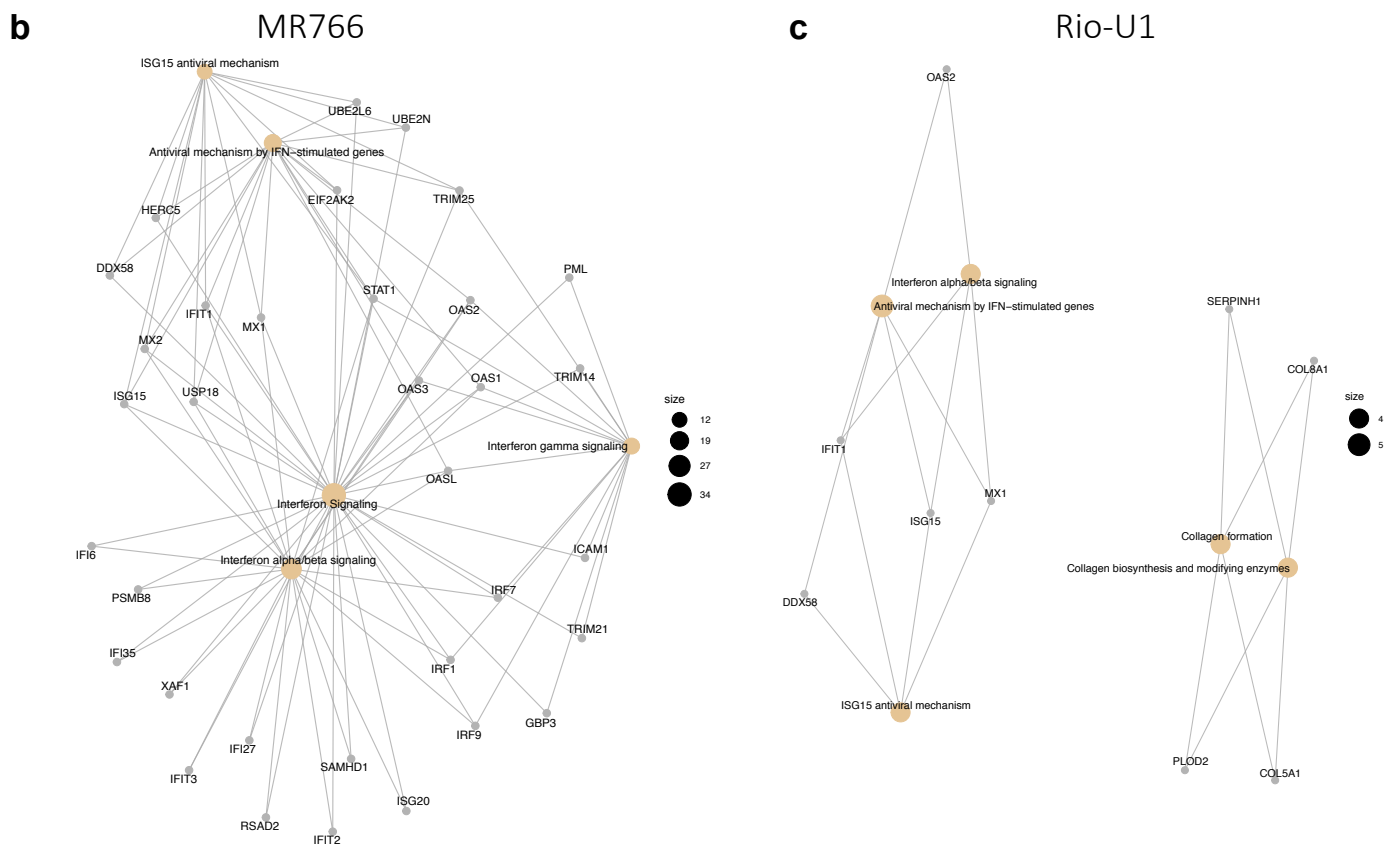

Supplement: Supplementary file 2 — Supplementary file2 - Supplementary Figure 2: Similarities in gene expression signatures (A) Bar graph showing the extent of up- and downregulation of genes that were similarly affected by either virus. Of the 186 genes that were differentially expressed by both viruses, the 50 genes with the highest level of significance in MR766 infected cells are shown. (B-C) Gene networks showing the overlap of genes involved in multiple signaling pathways in MR766 and Rio-U1 infected astrocytes. (PDF 365 KB) [file 13365_2020_931_MOESM2_ESM.pdf]

Figure S3

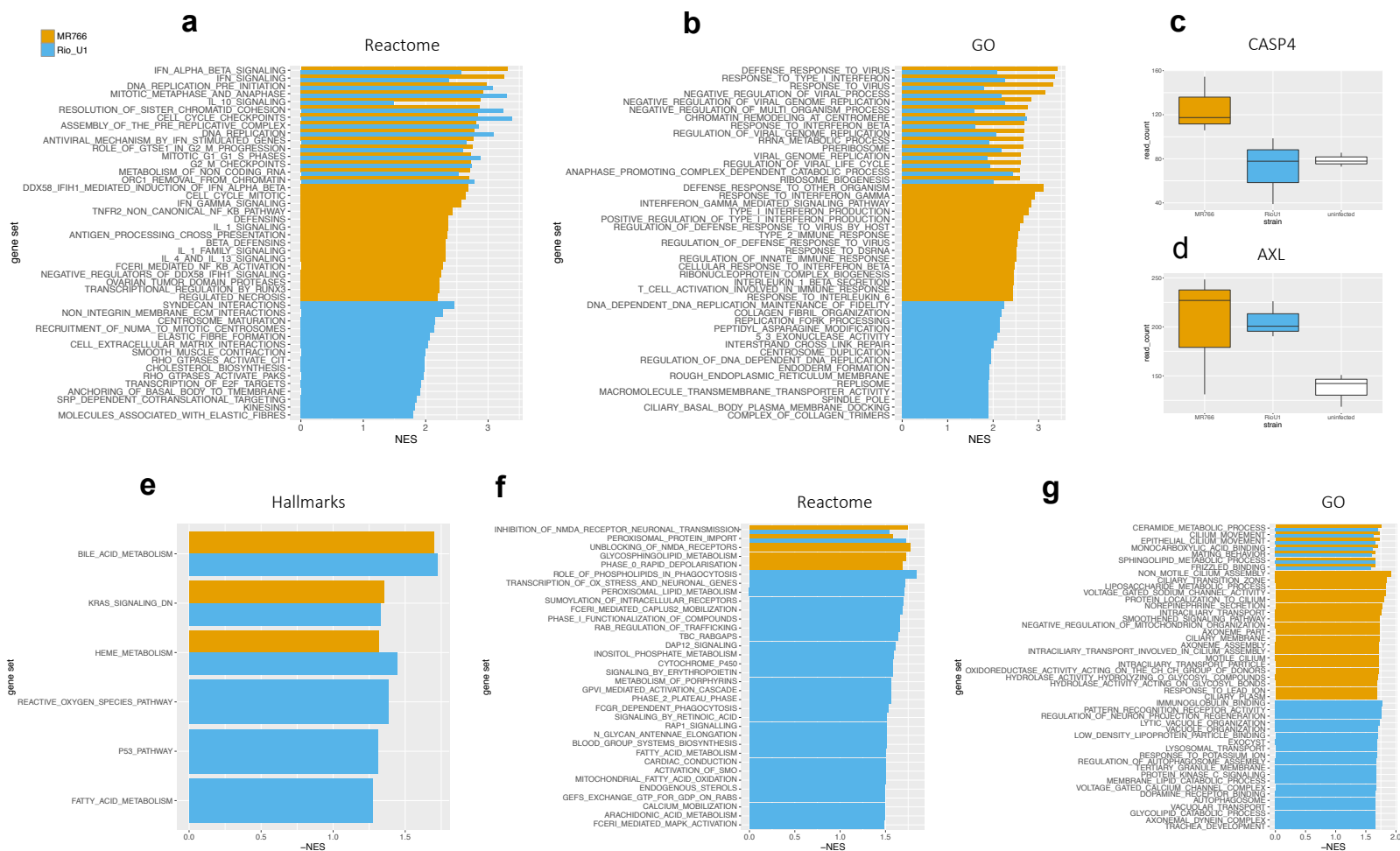

Supplement: Supplementary file 3 — Supplementary file3 - Supplementary Figure 3: Significantly modulated gene sets (A-B) Bar graphs showing gene sets in the Reactome (A) and Gene Ontology (GO) (B) collections that were significantly enriched at FDR<0.1. The top 10 gene sets that were either similarly enriched in both viruses (top) or enriched only in MR766 (middle, yellow) or Rio-U1 (bottom, blue) infected cells are displayed. (C-D) Boxplots showing normalized read counts for CASP4 (C) and AXL (D) in MR766 (yellow) or Rio-U1 (blue) infected cells and uninfected controls (white). (E-G) Bar graphs showing gene sets in the Hallmarks (E), Reactome (F), and Gene Ontology (GO) collections (G) that showed negative enrichment at FDR<0.25. (PDF 170 KB) [file 13365_2020_931_MOESM3_ESM.pdf]

Figure S4

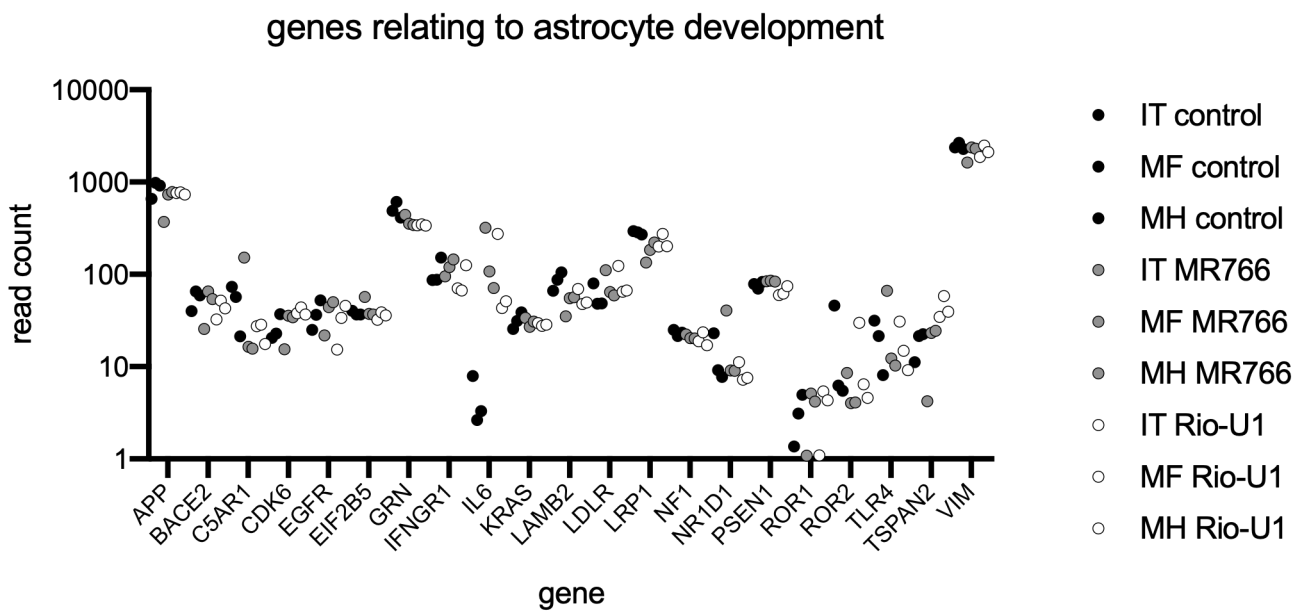

Supplement: Supplementary file 4 — Supplementary file4 - Supplementary Figure 4: Expression of genes relating to astrocyte developmentScatterplot showing the read counts of several genes relating to astrocyte development (GO:0014002). (PDF 168 KB) [file 13365_2020_931_MOESM4_ESM.pdf]
